# A Virtual Reality Video to Improve Information Provision and Reduce Anxiety Before Cesarean Delivery: Randomized Controlled Trial

**DOI:** 10.2196/15872

**Published:** 2019-12-18

**Authors:** Lore Noben, Simone Maria Theresia Anna Goossens, Sophie Eva Marieke Truijens, Marijn Marthe Georgine van Berckel, Christel Wilhelmina Perquin, Gerrit Dirk Slooter, Stefanus Johannes van Rooijen

**Affiliations:** 1 Department of Obstetrics and Gynecology Máxima Medical Center Veldhoven Netherlands; 2 Eindhoven MedTech Innovation Center Eindhoven Netherlands; 3 Department of Surgery Máxima Medical Center Veldhoven Netherlands; 4 Department of Anesthesiology and Pain and Palliative Care Máxima Medical Center Veldhoven Netherlands; 5 Care and Public Health Research Institute Maastricht University Medical Center Maastricht Netherlands

**Keywords:** virtual reality, cesarean section, counseling, preoperative care, surgery, anxiety, childbirth

## Abstract

**Background:**

Anxiety levels before cesarean delivery (CD) can lead to a negative birth experience, which may influence several aspects of the woman’s life in the long term. Improving preoperative information may lower preoperative anxiety and lead to a more positive birth experience.

**Objective:**

This study aimed to determine whether a virtual reality (VR) video in addition to standard preoperative information decreases anxiety levels before a planned CD.

**Methods:**

Women scheduled to undergo term elective CD were recruited from the outpatient clinic. They were randomized and stratified based on history of emergency CD (yes or no). All participants received standard preoperative information (folder leaflets and counseling by the obstetrician); the VR group additionally watched the VR video showing all aspects of CD such as the ward admission, operating theater, spinal analgesia, and moment of birth. The primary outcome measure was a change in score on the Visual Analogue Scale for Anxiety (ΔVAS-A) measured at admission for CD, compared with the baseline VAS-A score.

**Results:**

A total of 97 women were included for analysis. The baseline characteristics were similar in both groups, except for a significantly higher level of education in the control group. There was no significant decrease in the VAS-A score of the women in the VR group (n=49) compared with those in the control group (n=48; ΔVAS-A=1.0; *P*=.08; 95% CI −0.1 to 2.0). Subgroup analysis for the group of women with a history of emergency CD showed a trend toward decreased preoperative anxiety, despite the small sample size of this subgroup (n=17; *P*=.06). Of the 26 participants who provided completed questionnaires, 22 (85%) in the VR group reported feeling more prepared after seeing the VR video; of the 24 participants’ partners who completed the questionnaires, 19 (79%) agreed with the participants. No discomfort or motion sickness was reported.

**Conclusions:**

A VR video may help patients and their partners feel better prepared when planning a CD. This study showed that VR does not lead to a decrease in preoperative anxiety. However, subgroups such as women with a history of emergency CD may benefit from VR videos.

**Trial Registration:**

International Standard Randomised Controlled Trial Number (ISRCTN) 74794447; http://www.isrctn.com/ISRCTN74794447 (retrospectively registered)

## Introduction

### Background

A cesarean delivery (CD) is one of the most commonly performed surgeries in obstetrics, and the number of CDs performed is still increasing worldwide [1]. In 2017, 14% of all term deliveries in the Netherlands were performed via a CD, of which half were planned [2]. Data about the physical risks of the surgical procedure, such as infection and bleeding, are well known and should be part of the process of gaining informed consent. In the last few years, there is growing awareness about the psychological impact of CD [3].

The CD procedure is mostly performed under regional anesthesia, without sedatives or anxiolytics, to facilitate a conscious birth experience for the mother, prevent depression of the neonate, and promote immediate (skin-to-skin) contact between the mother and her baby [4].

Previous research demonstrated that the level of anxiety and fear of childbirth are known to be associated with the incidence of postpartum depression. Women who deliver by a CD are at risk for both increased fear of childbirth and postpartum depression [5]. It is essential to minimize preoperative anxiety for these patients because lower preoperative anxiety has been shown to lead to greater maternal satisfaction with CD and thus a more positive birth experience.

This is important because a negative birth experience is associated with serious negative long-term effects on several aspects of a woman’s life, such as the relationship with her partner and the baby and delay or even avoidance of future pregnancies [6,7]. Information provision has been shown to be a key element for quality of care, as perceived by women who gave birth [8].

Partners of women report anxiety and fear related to childbirth as well, especially with respect to a CD [9,10]. Providing both patients and their partners with good-quality information for CD might reduce preoperative fear and anxiety and thus improve satisfaction and recovery. Furthermore, it might minimize the possible negative long-term sequelae of a negative birth experience for both [11].

Currently, preoperative information is provided by the treating physician and through information folders. Ideally, a life guided tour and step-by-step explanation of the course of the procedure during the day should be part of the program for preparation. However, this elaborate preparation would require valuable time from the hospital personnel, and time is nowadays scarce and expensive. Owing to sterility issues, it is impossible to have a *life guided tour* through the operating room. Video education for surgery and medical interventions, as an alternative approach for a life guided tour, has been proven to improve immediate and short-term knowledge [12]. However, a difference in general anxiety (and anxiety and satisfaction with the consent process) is not uniformly shown [12]. Therefore, new technologies such as virtual reality (VR) may be of additional value, as patients can virtually experience the operating room and be better prepared for their surgery.

### Objectives

To our knowledge, this is the first study to investigate the effect of VR in addition to conventional information provision on the preoperative anxiety levels of women undergoing a planned CD. Our primary hypothesis was that adding the VR video to standard preoperative information would show a statistically significant decrease in preoperative anxiety compared with providing the standard preoperative information without a VR video. In addition, we expected a positive effect of VR on patients’ levels of anxiety and patient satisfaction scores of both women and their partners. Furthermore, this study aimed to determine whether VR would be feasible to implement, without causing any harmful side effects such as motion sickness.

## Methods

### Video Development

A total of eight women at the outpatient clinic who were scheduled to undergo a CD were interviewed. During this interview, we asked the women to describe their feelings in general and in terms of anxiety toward the planned CD as well as the way they received information about CD, their satisfaction with completeness of the information, and any possible improvements. We also asked for their opinion of the use of video and VR as possible information tools. All these women received information from their gynecologist and the internet, and one of them searched and watched a Web-based conventional video. Four women indicated that they would see additional value of information in a 2D video, whereas five women felt the same about VR. In addition, one woman indicated that she did not want to see a 2D video but was positive about a VR video because of the possibility of *watching away*. Of the women we interviewed, four did not expect a VR video to reduce their preoperative anxiety, three did not know what the effect of a VR video would be on their preoperative tension, and one thought a VR video might reduce preoperative stress. On the basis of this information, we developed a VR video, which can be viewed in the Infor-Med app (Infor-Med BV) on a smartphone or tablet.

**Figure 1:**
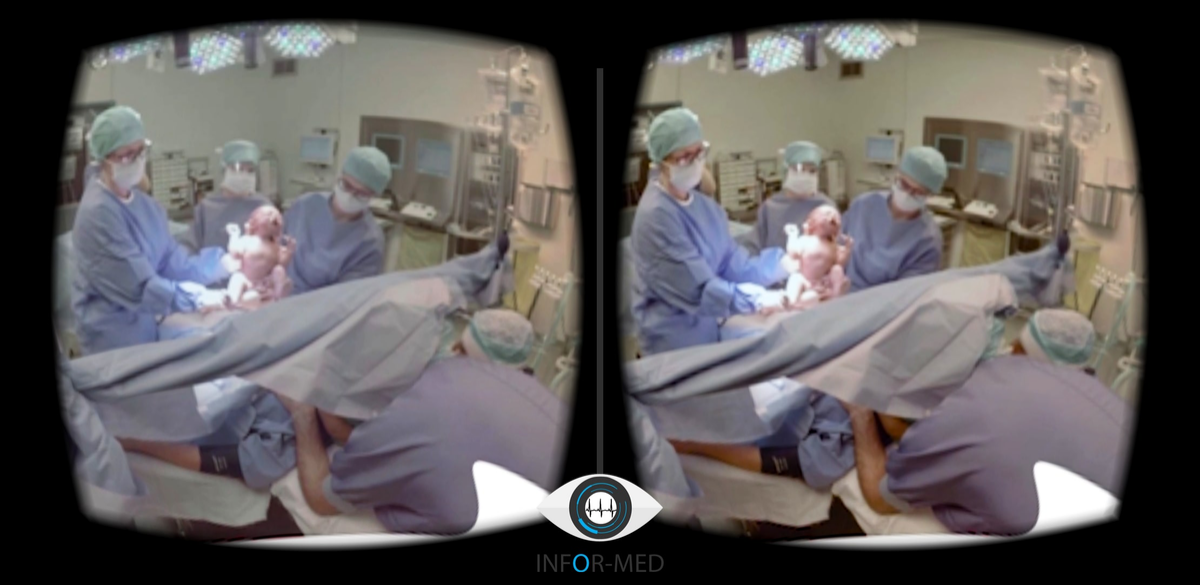
Screenshot from the 360 virtual reality video at the moment of birth.

The 360° VR video shows all the aspects of a CD, including the admission on the ward, the operating room, placement of spinal analgesia, and the birth of the baby when the gynecologist lifts the baby above the sterile environment (Figure 1). Instructions on viewing and downloading the VR video can be found on the website [13]. The video did not show any surgical content such as the area of incision. The video was recorded from the partner’s perspective in the operating room. The video ends at the ward, where the family is reunited. The video lasts 285 seconds and is narrated with a Dutch voice-over. Patients and their partners were continuously involved during the development of the video to allow us to include their feedback on the images, text, and changing the sequence.

### Study Design and Population

This randomized controlled trial included 80 women, enrolled from November 2016 to January 2018, who were scheduled for elective CD at Máxima Medical Center in Veldhoven, the Netherlands. We received a statement from our local institutional review board that no ethical approval was required (N17.017). Women were eligible for inclusion if they were aged 18 years or older, had planned for elective CD after 37 weeks of gestation, and had sufficient knowledge of the Dutch language. Exclusion criteria were prematurity (gestational age < 37 weeks), placenta previa, pre-eclampsia, and a suspected congenital anomaly. Patients were recruited from the outpatient clinic at our hospital. They were not explicitly informed that the study involved a VR video but were told that the intervention group received a novel method of information provision in addition to the standard information.

### Randomization and Masking

Randomization was performed by the researcher (LN) using a Web-based computer randomizer generating a randomization list. Couples were randomized into two groups by means of stratified block randomization: the control group received standard information from their doctor through information leaflets and oral counseling, and the intervention group (VR group) received the standard information and an additional VR video. Randomization blocks of 10 were used. Stratification was used based on the following two subgroups: (1) women with no history of CD and (2) women with previous emergency CD. The latter group was chosen, as these women may not have received elaborate information about the procedure before their first delivery because a vaginal delivery was intended. Masking of the researcher and participants was not possible because of the nature of the intervention.

### Procedures

**Figure 2:**
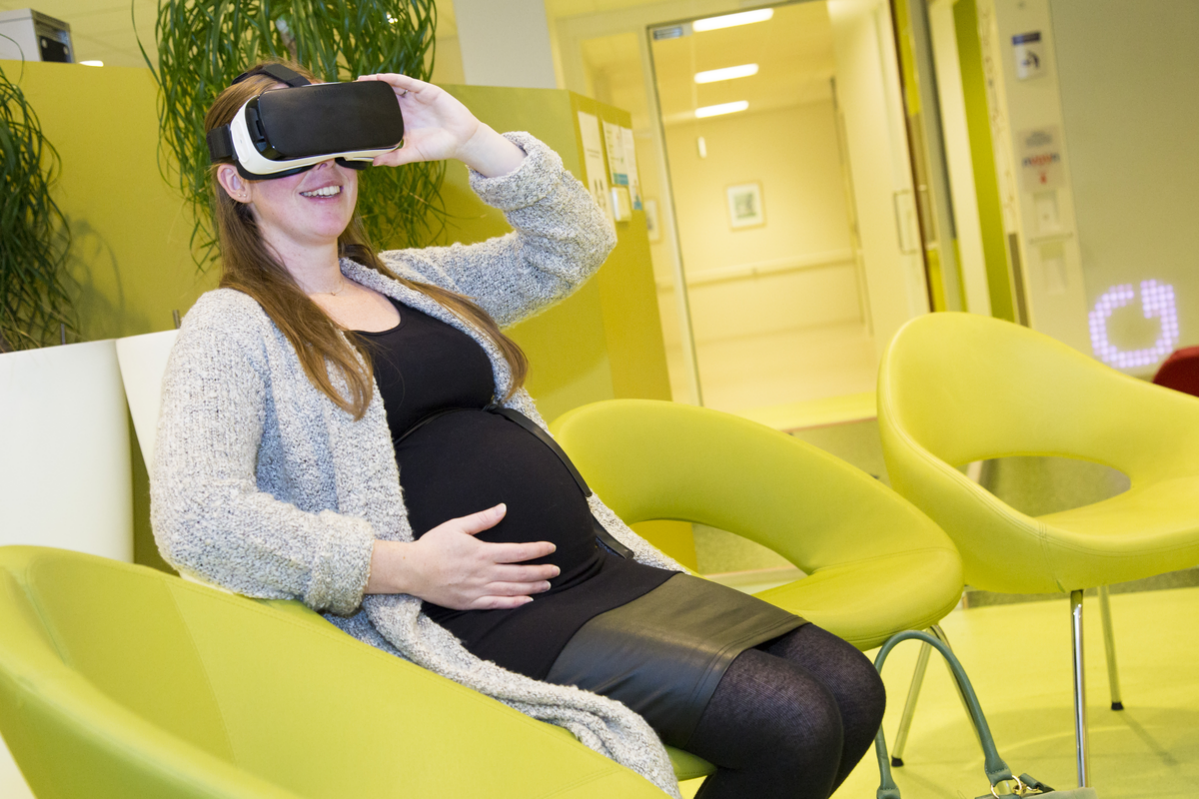
Illustration of the virtual reality glasses used.

**Figure 3:**
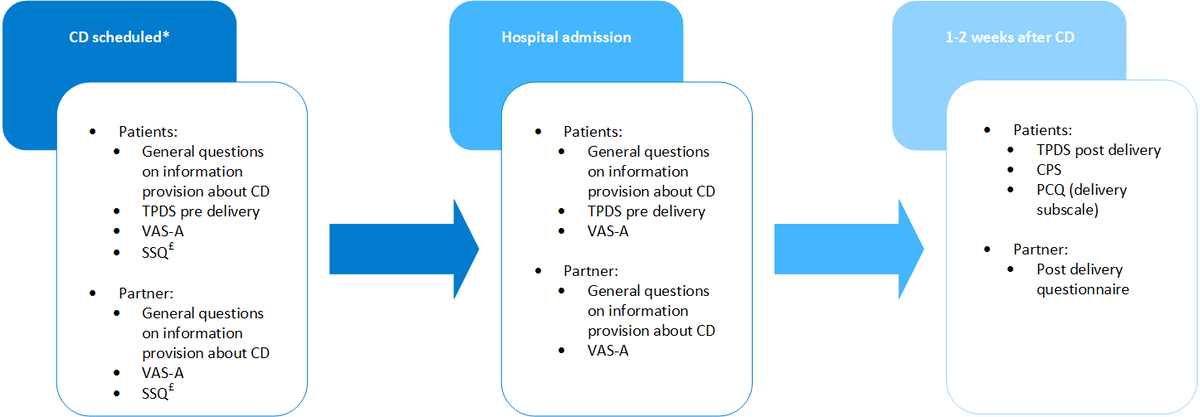
Time schedule and measured variables. *The intervention group additionally watched the 360° virtual reality video. £The intervention group additionally filled out the Simulation Sickness Questionnaire after watching the virtual reality video. CD: cesarean delivery; CPS: Childbirth Perception Scale; PCQ: Pregnancy and Childbirth Questionnaire; SSQ: Simulation Sickness Questionnaire; TPDS: Tilburg Pregnancy Distress Scale; VAS-A: Visual Analogue Scale for Anxiety.

After obtaining written informed consent, both women and their partners were asked to fill out the first questionnaire (time point 1). Subsequently, they were randomized into groups. If couples were randomized to the VR group, a VR video was shown using the Infor-Med app on the participant’s smartphone, and VR glasses (Figure 2) were supplied by the researcher at the outpatient clinic. Couples received a unique password to install and watch the video at the time of inclusion or later at home (unlimited views). By using a password, we prevented the possibility of patients in the control group gaining access to the VR video. The second questionnaire was filled out on the day of CD at admission to the ward (time point 2). If the woman was admitted earlier because of contractions, the questionnaire was filled out at that time. Couples were asked to fill out the third questionnaire 1-2 weeks after CD (time point 3). The questionnaire time schedule is shown in Figure 3. The primary outcome measure was the mean score on the Visual Analogue Scale for Anxiety (VAS-A) at hospital admission (time point 2). Data on the baseline characteristics were collected from the electronic patient file.

### Questionnaires

The Simulation Sickness Questionnaire (SSQ) with 13 questions regarding the symptoms related to motion sickness was added to the first questionnaire for the VR group [14]. These symptoms were scaled from none (1) to severe (4).

The VAS-A was used in the first and second questionnaires to measure preoperative anxiety [15]. It comprises a 10-cm horizontal line, stating, on the left end, “not anxious at all” and, on the right end, “most anxious I can imagine.”

The Tilburg Pregnancy Distress Scale was used in the first and second questionnaires. This questionnaire consists of 16 items regarding the woman’s perception of her pregnancy, divided over two subscales: negative affect and partner involvement. Questions are formed in positive and negative statements. Items were recoded such that a higher score represents a higher level of distress [16]. The Childbirth Perception Scale (CPS) was used in the third questionnaire, which was filled out approximately 1 week after the CD. The CPS consists of 12 items with a *perception of delivery* subscale (6 items) and a *perception of the first postpartum week* subscale (6 items). Items were recoded such that a higher score corresponded with more distress and less positive perception [17]. Women were also asked to fill out the *pregnancy* subscale of the Pregnancy and Childbirth Questionnaire (PCQ) in the third questionnaire, consisting of seven items. Items were recoded such that higher scores indicate higher quality of care. This third questionnaire also contained a short questionnaire with five items for the partners, regarding their experience of this postpartum week. A translated version of this short questionnaire is presented in Multimedia Appendix 1. Furthermore, we asked participants if the preoperative information was sufficient. Participants in the VR group received the additional question if they felt more prepared for CD after seeing the VR video (response: yes or no).

### Statistical Analyses

Sample size calculation was performed using the software G*power3 [18] and based on VAS-A scores from the literature [19]. Given a mean VAS-A score of 5.01 (SD 3.14) cm [19], 38 patients were included in each study arm to detect a decrease in VAS-A score of 2 cm. A sample size of 38 patients per study arm was determined based on detecting a decrease in VAS-A of 2 cm, with 80% power and a significance level of .05. To account for missing data, we set the sample size at 40 inclusions per study arm.

Statistical analyses were performed using SPSS (version 25; IBM Corporation, Armonk, New York). To test for differences in baseline characteristics between the two groups, a Student *t* test and Mann-Whitney *U* test were used for normally and nonnormally distributed data, respectively. For both women and their partners, the difference in VAS-A score at the second time point between the VR group and the control group was calculated using a Student *t* test, as these data were distributed normally. To test the influence of the VR video on the change in VAS-A score between the first and second time points for each study group, a linear regression analysis was performed. Thereafter, the history of CD was added as an independent factor in a multivariate regression analysis. The following variables were found to have an influence on the VAS-A score in previous literature: baseline VAS-A (time point 1), a psychiatric history of depression or anxiety disorder, level of education, previous CD, age, and marital status. Subsequently, randomization to the VR group was added as an independent factor in multivariate regression analysis for each variable that showed a significant contribution to the regression model.

As the history of emergency CD is known to be a predictor of preoperative anxiety, we performed a separate regression analysis to determine if there was a significant effect of the VR video in this subgroup alone. This was possible because of the stratified randomization applied.

For the same reason, differences in scores on the questionnaires were calculated separately for both the groups with and without a history of emergency CD. After recoding, the scores of the individual items were added to obtain an overall score. We calculated the difference between this overall score for each of the questionnaire subscales between the VR group and the control group using a Student *t* test.

## Results

### Participant Characteristics

**Figure 4:**
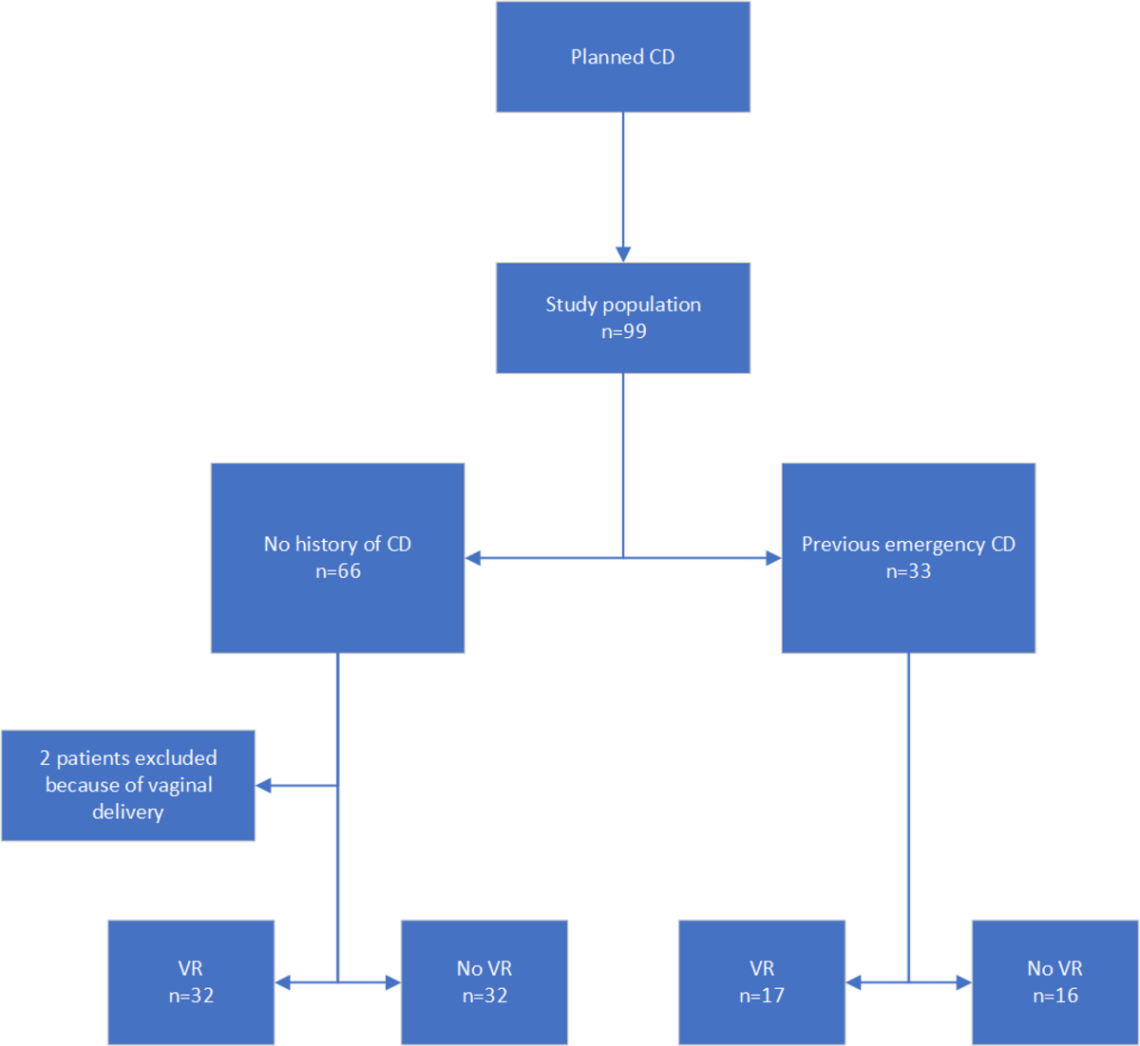
Flowchart of patient inclusion. CD: cesarean delivery; VR: virtual reality.

A total of 99 patients were included. Owing to the high number of missing questionnaires at the start of the study, we decided to continue including patients until we reached 80 completed questionnaires at time point 2, which included our primary outcome measure. Moreover, two patients were excluded because of spontaneous vaginal delivery after randomization. From the remaining 97 patients, we received 94% (91/97) completed questionnaires at time point 1, 87% (84/97) completed questionnaires at time point 2, and 73% (71/97) completed questionnaires at time point 3. Figure 4 shows the flowchart of patient inclusion.

Baseline characteristics for both the VR group and the control group are shown in Table 1. There were no differences between the groups with respect to age, gestational age at delivery, parity, and the incidence of previous CD. We noticed a significant difference in the level of education, with a higher proportion of participants with a high level of education level in the control group (*P*=.03).

**Table 1:** Baseline characteristics of women undergoing cesarean delivery for both study groups (probability values were calculated using an independent *t* test for normally distributed data; otherwise, a Mann-Whitney *U* test was used).

Characteristics			Virtual reality group (n=49)		Control group (n=48)
Age (years), mean (SD)			32.6 (3.9)		33.12 (4.3)
Gestational age (weeks) on delivery, mean (SD)			39.0 (0.7)		38.8 (0.8)
Gravidity, median (IQR^a^)			2 (1-3)		2 (1-2)
Parity, median (IQR)			1 (0-1)		1 (0-1)
Previous CD, n (%)			17 (35)		16 (33)
**Marital status, n (%)**					
	Married	30 (61)		24 (50)	
	Living together	15 (31)		19 (40)	
	Single	0 (0)		2 (4)	
	Missing	4 (8)		3 (6)	
Body mass index (kg/m^2^), median (IQR)			24.8 (22.9-29.2)		24.9 (22.2-27.5)
**History of depression or anxiety, n (%)**					
	Yes	13 (27)		10 (21)	
	No	33 (67)		35 (73)	
	Missing	3 (6)		3 (6)	
**Level of education^b^, n (%)**					
	University/college	24 (49)		32 (67)	
	Secondary education	0 (0)		3 (6)	
	Vocational training	17 (35)		8 (17)	
	Prevocational education	5 (10)		2 (4)	
	No education or primary education	0 (0)		0 (0)	
	Missing	3 (6)		3 (6)	
**Indication for CD^c^, n (%)**					
	Repeat CD	14 (29)		16 (33)	
	Fetal breech position	24 (49)		17 (35)	
	History of obstetric complications	5 (10)		8 (17)	
	Current obstetric complications	0 (0)		2 (4)	
	Medical history	4 (8)		2 (4)	
	Patient’s request	2 (4)		3 (6)	
**Information sources, n (%)**					
	Health care professional	38 (78)		36 (75)	
	Patient folders	37 (76)		40 (83)	
	Experiences from friends/family	24 (49)		27 (56)	
	Internet	25 (51)		27 (56)	
	YouTube	8 (16)		10 (21)	
	Other	8 (16)		6 (13)	

^a^IQR: interquartile range.

^b^Significant difference (*P*<.05) in ranks between the VR group and the control group as calculated with the Mann-Whitney *U* test.

^c^CD: cesarean delivery.

### Primary Outcome Measures

There was no significant difference in the mean VAS-A score at admission between the control group and the VR group for both women (4.6 [SD 2.5] vs 5.6 [SD 2.4], respectively; *P*=.08) and their partners (3.4 [SD 2.0] vs 3.9 [SD 2.5], respectively; *P*=.30). There was no difference between the control group and the VR group in terms of baseline VAS-A scores for the women (3.8 [SD 2.3] vs 4.1 [SD 2.3], respectively; *P*=.52) or their partners (2.5 [SD 1.9] vs 2.5 [SD 2.3], respectively; *P*=.98). There was an increase in the VAS-A score (ΔVAS-A) between the first and second measurements of 1.5 cm for the women in the VR group compared to 0.8 cm for women in the control group (95% CI −0.1 to 2.0; *P*=.08). For their partners, there was an increase of 1.4 cm in the VR group compared to 0.9 cm in the control group (95% CI −0.5 to 1.6; *P*=.30). Table 2 gives an overview of these results.

**Table 2:** Difference in the Visual Analogue Scale for Anxiety value at time point 2 (at admission) between the virtual reality and the control groups for both women and their partners (difference calculated using the Student *t* test).

Study arm		VAS-A^a^ Q1^b^, mean (SD)		VAS-A Q2^c^, mean (SD)		Change in score on the Visual Analogue Scale for Anxiety		Mean difference^d^		*P* value^d^		95% CI^d^	
**Women**								1.0		.08		−0.1 to 2.0	
	Control group		3.8 (2.3)		4.6 (2.5)		0.8						
	VR^e^ group		4.1 (2.3)		5.6 (2.4)		1.5						
**Partners**								0.6		.30		−0.5 to 1.6	
	Control group		2.5 (1.9)		3.4 (2.0)		0.9						
	VR group		2.5 (2.3)		3.9 (2.5)		1.4						

^a^VAS-A: Visual Analogue Scale for Anxiety.

^b^Q1: questionnaire time point 1.

^c^Q2: questionnaire time point 2.

^d^These values are given for the change in score on the Visual Analogue Scale for Anxiety between the control group and the VR group for both women and their partners.

^e^VR: virtual reality.

The following variables showed a significant relation with ΔVAS-A: baseline VAS-A (*F*_1,75_=8.4; *P*=.01) and history of CD (*F*_1,75_=6.0; *P*=.02). These variables were incorporated in our multivariate regression, which significantly explained 16% of the variance in change in VAS-A score between time points 1 and 2.

As we used stratified randomization, we were able to perform a separate analysis based on the history of CD using an interaction term. Increase in the baseline VAS-A score at time point 2 (at admission) in women in the VR group with a history of emergency CD was 1.7 cm smaller than that in women with a history of emergency CD in the control group, although this effect was not significant (*P*=.06).

As there was a baseline difference in the level of education between both groups, we performed a regression analysis to analyze the effect of the different groups on the difference in VAS-A score, based on their level of education. By using dummy variables with university/college as a reference group, we were able to perform regression analysis on the categorical variables. If secondary education was the reference group, women had slightly lower VAS-A scores (0.2 cm). When vocational training or prevocational education was the reference group, the VAS-A scores were higher (0.5 and 0.2 cm, respectively). However, these results were not significant.

### Secondary Outcome Measures

Median scores on the SSQ for motion sickness symptoms ranged from 1.0 to 1.5, reflecting the absence of discomfort caused by the VR video.

There was no significant difference in scores on the Tilburg Pregnancy Distress Scale subscales for both time points 1 and 2 between the VR group and the control group. The negative affect subscale showed higher scores at time point 2 than at time point 1 (control group, 25.2 [SD 3.9] vs 9.4 [SD 4.9]; VR group, 23.8 [SD 4.4] vs 9.1 [SD 4.4]), but this increase was equally present in both study arms.

For the PCQ questionnaire after delivery, we found a significantly higher score for the VR group without a history of emergency CD, indicating that they perceived a higher quality of care than the control group (10.2 [SD 3.8] vs 12.9 [SD 3.5]; *P*=.02). There was no significant difference between the control group and VR group of women with no history of emergency CD.

We received 26 completed questionnaires from time point 3 from women in which the question regarding the additional value of the VR video was filled in. Of the 26 women, 4 (15%) responded that they did not feel more prepared after seeing the VR video. The remaining 22 (85%) women responded positively. From the partners, 24 questionnaires were completed from time point 3 including this question, of which 19 (79%) partners responded positively. The remaining 5 (21%) partners did not feel more prepared after seeing the VR video.

## Discussion

### Principal Findings

In this study, we aimed to assess the effect of adding a VR video as part of standard preoperative information before planned CD on the information level of women and their partners and the woman’s level of anxiety. Our data showed that VR is not related to a significant decrease in self-reported preoperative anxiety for both women and their partners compared with a no VR condition. Only women with a history of emergency CD showed a trend toward decreased preoperative anxiety. With regard to the quality of care, women and their partners perceived a higher quality of care after watching the VR video.

### Comparison With Previous Studies

Our preoperative VAS-A scores match those previously described in the literature [19,20]. As expected, anxiety levels were higher on the day of planned CD than at the baseline for both women and their partners, independent of the study arm. Differences in baseline characteristics of both groups, despite the randomized design of our study, might have influenced these results. Baseline anxiety level is an important predictor for preoperative anxiety, as stated in the literature and confirmed in our data. Baseline anxiety scores were slightly higher in the VR group compared with the control group for women in our study group, although this difference was not significant. In addition, there was a difference in the level of education between our VR and control groups at baseline, with the latter containing more women with a higher level of education. A higher level of education has previously been shown to be associated with increased preoperative fear [21]. Baseline anxiety scores were (nonsignificantly) higher in the VR group, although we would have expected the opposite based on the level of education. We could not reproduce this effect in our data through regression analysis. These baseline differences, therefore, probably did not significantly influence our primary outcome measure.

Besides the sociodemographic features (level of education and marital status), other factors such as presence of previous psychiatric disorders are known to influence preoperative anxiety. Women with a history of anxiety are more likely to experience fear of childbirth than others [22-25]. We could not reproduce this effect in our results. However, the percentage of women with a history of anxiety or depression included was low in both groups.

Another known risk factor for fear of childbirth is previous emergency CD [6]. In the case of emergency CD, women are rushed to the operating room because of complications that arise during labor, which creates a stressful situation. In the group of women with a history of emergency CD, adding the VR video to the standard preoperative information led to a 1.7 cm smaller increase in ΔVAS-A, with a *P* value at the border of significance (*P*=.06). However, given our cutoff value of 2 cm, we consider this result as not clinically relevant. Previous studies also reported lower preoperative anxiety in case of prior exposure to surgery [20,21,26,27]. Unfortunately, we have only taken into account previous (unplanned) CD and did not look at the history of surgery, in general, as a risk factor. Future studies should consider assessing for this confounder.

Literature about the use of (virtual) information videos in patient counseling report conflicting results of their effect on preoperative anxiety [28-33]. Anxiety is a multifactorial phenomenon, making it impossible to account for each interpersonal difference. Differences in study design concerning correction for confounding risk factors may influence the results. During the last two decades, there has been an increase in interest in the psychological impact of preoperative anxiety, both in general surgery and obstetrics. Besides (virtual) information videos, other methods to decrease preoperative anxiety have been studied, such as acupressure, intraoperative music therapy, and various information platforms [34-36]. VR has a big advantage of providing a visual reality–based experience. It offers a sense of *having been there*. This might help patients adjust their expectations toward the operation [37]. Furthermore, VR videos can be made in different languages very easily, and videos, in general, may be more accessible than written information for people with low literacy. However, as VR is a multisensory experience, motion sickness can occur in users. These complaints are mostly short lived but can cause real discomfort for users [38]. No cases of motion sickness occurred during our study after watching the VR video.

### Recommendations

Although there was no significant difference in the main outcome measure, we found a trend toward decreased preoperative anxiety in the subgroup of women with a history of emergency CD after watching the VR video. This indicates that this subgroup of patients may benefit from this method of preoperative information. Careful selection of subgroups is the next step before implementing this information medium as part of standard care. Offering the VR video without obligation as part of the preoperative information at the outpatient clinic could help in gaining insight into the target population. Through tracking which and how many patients watch the VR video, it may be possible to assess the characteristics of this patient population interested in the video. However, patient privacy regulations make it difficult to facilitate this. Anonymous feedback questions provided through the mobile app after watching the video may add in retrieving this information. In addition, in the group of patients who waive the possibility of watching the VR video, it is important to ask for their reasons, which can be used in optimizing the content of the VR video.

### Strengths and Limitations

With the rise of this innovative modality, a guideline toward the setup of clinical studies concerning the use of VR has recently been published. The authors recommend a three-phase development and validation process to uniformize the development and validation of VR applications [39]. Although our study was conducted before these guidelines were published, we believe our study comprises elements from all three phases suggested in the guidelines: We involved patients in the development process of the VR video (VR 1 phase), we conducted a randomized controlled trial to study the effect of VR on preoperative anxiety (VR 3 phase), and we briefly asked participants about their experience with the VR video (VR 2 phase). For future studies, adherence to these guidelines is desirable to ensure uniformity within the VR science platform.

There are several limitations to this study. First, power calculations were made based on the primary outcome measure (preoperative VAS-A score). Therefore, this study may be underpowered to show significant differences in results from the questionnaires designed to measure psychological functioning in the perinatal period. The only significant result was a higher score on the PCQ questionnaire for the VR group without a history of emergency CD, indicating that they perceived a higher quality of care than the control group. However, the absolute difference between both groups is small and therefore not clinically significant. The relatively small study population is a limitation for each of the subanalyses performed in our study.

Second, we did not keep track of the number of people who refused to participate in the study and their arguments for refusal. This may have led to a selection bias because patients who are not keen on watching the VR video may be more likely to refuse when asked to participate. In addition, we did not verify if all participants who were randomized to the VR group actually watched the entire VR video. Although most participants watched the VR video at the time of inclusion, some chose to watch the video at home in their own environment. Therefore, there is a chance that some of them did not see the video, and including these completed questionnaires in our analysis could have caused a bias.

Fourth, there was a high amount of missing data because of missing questionnaires. We distributed paper questionnaires to patients at the time of inclusion and placed duplicate questionnaires at the ward where patients were admitted for CD. The researchers actively pursued the questionnaires. Despite our efforts, the percentage of missing questionnaires remained high, especially for questionnaire form 3. In future studies, electronic questionnaires available through a mobile app on a smartphone could facilitate this process.

### Conclusions

Our study did not show a decrease in preoperative anxiety after VR information provision for patients undergoing elective CD. There was a trend toward decreased preoperative anxiety in the subgroup of women with a history of emergency CD who watched the VR video. Further research for identifying the characteristics of subgroups of patients who would potentially benefit from VR information provision is necessary.

## Appendix

Multimedia Appendix 1 Translated version of the postdelivery questionnaire for partners (questionnaire 3).

Multimedia Appendix 2 CONSORT-EHEALTH checklist (V 1.6.1).

## Abbreviations

CD: cesarean delivery
CPS: Childbirth Perception Scale
PCQ: Pregnancy and Childbirth Questionnaire
SSQ: Simulation Sickness Questionnaire
VAS-A: Visual Analogue Scale for Anxiety
VR: virtual reality
ΔVAS-A: change in score on the Visual Analogue Scale for Anxiety
