# Supplementary material for: A Virtual Reality Video to Improve Information Provision and Reduce Anxiety Before Cesarean Delivery: Randomized Controlled Trial
Source: JMIR Ment Health. 2019 Dec 18;6(12):e15872. doi: 10.2196/15872 (PMC6939281; doi:10.2196/15872)
Supplement: Multimedia Appendix 1 [file mental_v6i12e15872_app1.docx]

**Questionnaire 3 (Partner)**: 1 week after childbirth

Date:

|  |  | Totally agree | Agree | Neutral | Disagree | Totally  Disagree |
| --- | --- | --- | --- | --- | --- | --- |
| 1 | I feel that my partner and I experience the development process of the baby together. |  |  |  |  |  |
| 2 | I feel that I can discuss my feelings and concerns relating to the development and care of the baby with my partner |  |  |  |  |  |
| 3 | I feel like I stand alone in taking care of the baby |  |  |  |  |  |
| 4 | The birth of our baby has brought me and my partner closer together |  |  |  |  |  |
| 5 | I feel supported by my partner |  |  |  |  |  |
